# Protocol for incomplete murine tumor resection to study local effects of surgical trauma on residual sarcoma

**DOI:** 10.1016/j.xpro.2026.104481

**Published:** 2026-03-31

**Authors:** Tyler R. McCaw, Hy B. Dao, Sin Yee (Amelie) Lim, Stuart A. Fine, Michael S. Shehata, Fritz C. Eilber, Joseph G. Crompton

**Affiliations:** 1 Department of Surgery, Division of Surgical Oncology, University of California, Los Angeles, Los Angeles, CA, USA; 2 Broad Center of Regenerative Medicine and Stem Cell Research, University of California, Los Angeles, Los Angeles, CA, USA; 3 David Geffen School of Medicine, University of California, Los Angeles, Los Angeles, CA, USA; 4 Oregon Health & Science University School of Medicine, Oregon Health & Science University, Portland, Portland, OR, USA; 5 Jonsson Comprehensive Cancer Center, Sarcoma Oncology Program, University of California, Los Angeles, Los Angeles, CA, USA

**Keywords:** Cancer, Health Sciences, Model Organisms

## Abstract

Oncologic surgery aims to remove the entirety of cancer from a patient. However, we have little understanding of how surgical trauma impacts the residual primary tumor when disease is often left behind. Herein, we present a protocol to test the impact of surgical trauma on an incompletely resected murine sarcoma. We describe steps for tumor cell injection, tumor measurements, experimental group assignments, and maintaining sterility. We then detail procedures for surgical preparation, incision and tumor exposure, incomplete tumor resection, closure, and recovery.

## Highlights

• Protocol to study effects of incomplete sarcoma resection
• Steps to generate a primary tumor and perform incomplete resection survival surgery
• Detailed instructions regarding surgical technique for reproducible results

## Abstract (editor highlights)

Publisher’s note: Undertaking any experimental protocol requires adherence to local institutional guidelines for laboratory safety and ethics.

## Graphical abstract

**Figure:**
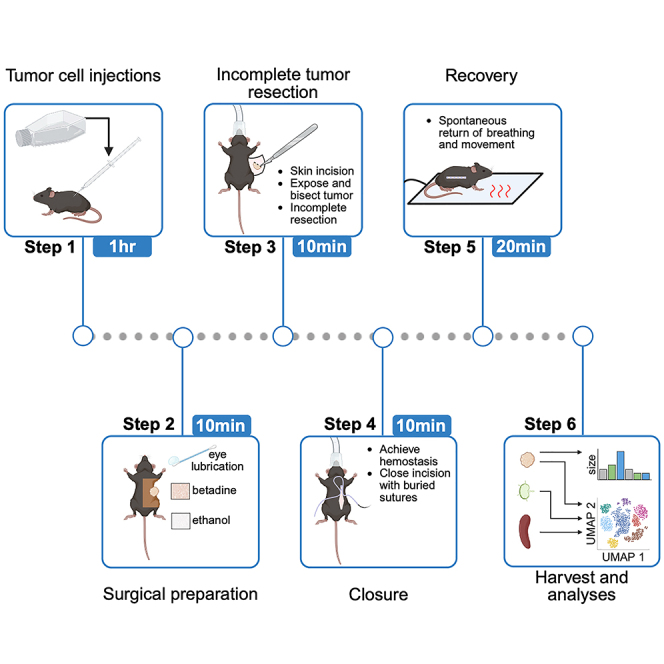

## Before you begin

### Incomplete oncologic resection model

The intent of curative oncologic surgery is to remove 100% of cancer; however, this is not always possible. Understanding how surgical trauma affects residual cancer (or cancer remaining in the body after surgery) represents a key knowledge gap in clinical oncology. In the case of microscopic residual cancer, surgeons remove all visible disease, but post-operative pathology review reveals microscopic deposits extending to the resected tissue margin. Gross or visible residual cancer may be encountered due to (i) interrelatedness of tumor with critical anatomic structures or (ii) initial misdiagnoses of a mass leading to early termination of the surgery and referral to a more experienced center. Indeed, among the 10 most common solid cancers in the United States, approximately 5–35% of surgeries will have positive surgical margins (i.e., local, residual primary tumor after surgery).^1^ Although a relatively common clinical scenario, our understanding of how surgery itself impacts residual primary tumors has thus far been limited by the distinct lack of a reproducible experimental model that faithfully recapitulates local surgical trauma.

### Innovation

Previously reported models of surgical trauma have included implantation of a sterile sponge to promote a wound healing response, which in some ways resembles chronic tumor inflammation.^2^^,^^3^ Complete tumor excision has been used in metastatic models.^4^^,^^5^^,^^6^^,^^7^^,^^8^ However, this approach suffers from frequent, yet inconsistent, local tumor recurrence, impacting metastatic growth and mouse survival. Moreover, surgery itself promotes metastatic progression,^9^ thus, altering the native cancer biology and disease trajectory. Both approaches are limited by an inability to study the impact of surgical trauma on a residual, local tumor microenvironment. Laparotomy with subsequent study of tumors at various sites has also been used^10^^,^^11^ and enables study of systemic surgical effects, but not local effects of surgical trauma.

In contrast, the novel model of incomplete primary tumor resection described herein enables study of local effects of cancer surgery (i.e., cancer remaining at the surgical resection site). The protocol was developed using TAO1 murine sarcoma cells; however, we have also readily adapted it to various other cancer cell lines, including B16F10, LLC, KPC. In addition to the incomplete tumor resection group, control groups should include tumor-bearing mice undergoing no surgery and sham surgery (an incision of equal length but on the contralateral flank, away from the tumor). In this way, systemic effects of surgery (sham group) can be distinguished from local effects (incomplete tumor resection group). Thus, the model of incomplete oncologic murine surgery described herein is critical to advancing the field’s understanding of how surgery itself impacts residual cancer.

### Mouse strains

All experiments are performed using mice of at least 6–8 weeks old, but no older than 6 months. Immune competent, wild-type B6 mice were ordered from Jackson Labs. Female and male mice use should ideally reflect gender bias (or lack thereof) in the corresponding human cancer being modeled. Mice were housed in the University of California, Los Angeles animal facility at 65–75F with 40%–60% humidity. Mice were allowed to acclimate for 3–7 days prior to use in experiments (per institutional practice). Animal husbandry was provided by the Division of Laboratory Animal Medicine (DLAM).

### Tumor cell maintenance

1. Passage thawed TAO1 tumor cells a minimum of two times and no more than seven times prior to injection.
2. Culture TAO1 cells in media containing 84% DMEM 1X (Gibco), 15% FBS, and 1% penicillin/streptomycin at 37C and 5% CO_2_.
3. Perform mycoplasma testing every 6 months.

### Tumor cell injections

**Timing: Cell harvest: 45**–**60 min, cell injection: 2 min per mouse**

4. The day prior to tumor cell injection, shave the right flank of each mouse from hind leg to foreleg and about 1.5–2 cm wide using clippers.
5. Harvest tumor cells between 70%–90% confluence using 0.25% trypsin.
  a. Wash adherent cells twice with 1X PBS,
  b. Add a sufficient volume of 0.25% trypsin to coat the entire surface of the culture dish by rocking back and forth, approximately 2 mL for a T75 flask.
  c. Place the culture dish in a 37C incubator for 4 to 5 min.
  d. Remove culture dish and add FBS-containing media to effectively “quench” the action of trypsin and rinse cells off the surface of the culture dish.
  e. Collect cells in a 15 mL conical.
  f. Add 1X PBS to a total volume of 10 mL.
6. Wash tumor cells in 1X PBS two more times.
  a. We centrifuge cells at 500 *g* for 5 min, but other centrifuge settings may appropriate for cells being used.
  b. Resuspend cells in 10 mL PBS.
  c. Repeat steps 6a and 6b for a total of two washes.

***Optional:*** Depending on the tendency of cell line being used to form clumps, consider passing cells through a 40 μm filter and slowly pass through a 25G needle attached to a 3 mL syringe 2 to 3 times.

7. Count and dilute cells to an appropriate working concentration in PBS.
  a. Count cells using a hemocytometer or an automated cell counter.
  b. Dilute cells in 1X such that the desired number of cells for injection is present in 50 μL. For TAO1 cells, we inject 1.5E5 cells per 50 μL or 3.0E6 cells per mL.

***Note:*** The concentration of cells per 1 mL is then equal to total number of cells per injection × 20.

8. Maintain cells on ice until injection.
  ***Note:*** The decision to use Matrigel (or similar basement membrane matrices) for injections, or not, must be weighed against the variable presence of growth factors in different formulations.^12^ We describe tumor cell injection without Matrigel in this protocol.

9. Immediately prior to tumor cell injection, anesthetize mice in an isoflurane chamber.
10. Quickly clean the right flank with an ethanol wipe.
11. Prior to injecting cells, briefly vortex to ensure even distribution of cells prior to drawing up in 0.5 mL insulin syringe.
12. Inject 50 μL of prepared cells into the subcutaneous flank.

***Note:*** Take care to place cells more ventrally than dorsally on mouse flank. Inject cells closer to the hind leg (but away from inguinal lymph node) than foreleg (at approximately 30%–40% of the distance from hind leg to foreleg) such that an incision can be made with sufficient space for future tumor exposure and dissection.

### Institutional permissions

Ensure murine surgery on tumor-bearing mice is adequately described and has received approval through your home institution’s regulatory bodies. The surgical protocol herein was approved by the University of California, Los Angeles Animal Research Committee and Institutional Animal Care and Use Committee (ARC-2023-064).

## Key resources table

**Table:** 

REAGENT or RESOURCE	SOURCE	IDENTIFIER
**Chemicals, peptides, and recombinant proteins**		

Phosphate buffered saline (PBS) pH 7.4 (1X) without calcium, magnesium	Gibco	14040216
0.25% trypsin∗	Gibco	25200056
Carprofen (1 mg/cup)	Med Vet International	RXRIMINJ
Medigel sucralose	Vivarium	74-02-5022
10% betadine solution	Med Vet International	OTCBSO16P
70% ethanol	Fisher Scientific	11-002-215
Isoflurane∗	VetOne	502017

**Experimental models: Cell lines**		

TAO1 (Trp53^fl/fl^; Kras^G12D/+^, lenti-cre injection into muscle, C57BL/6 background, female, 6–8weeks old)∗	Laboratory of Michael Monument	Hildebrand et al.^13^

**Experimental models: Organisms/strains**		

C57BL/6J mice (male and female, 6–8 weeks old)	Jackson Labs	Strain# 000664; RRID: IMSR_JAX:000664

**Other**		

Clippers∗∗	Fisher Scientific	NC0154323
15 mL conical	Genesse	28-103
40 μm filter∗∗	Fisher Scientific	22-363-547
25G × 1 1/2 needle∗∗	Med Vet International	305125
3 mL plastic syringe∗∗	Fisher Scientific	14955457
Induction chamber∗	Patterson Scientific	78933387
Charcoal canister	Am Bickford	80120
Isoflurane vaporizer∗	Vivarium	N/A
Oxygen tank	Vivarium	N/A
Ethanol wipes∗∗	Fisher Scientific	22-363-750
Table top vortex∗∗	Fisher Scientific	14-955-151
0.5 mL insulin syringe∗	VetOne	510107
Calipers	Fisher Scientific	15-077-958
Absorbant underpad∗	Fisher Scientific	14-206-65
Boncare heating pad∗∗	Amazon	N/A
2″ × 2″ gauze∗	Fisher Scientific	22-362178
GenTeal tears (eye lubrication)	Med Vet International	1153231EA
Q-tips∗∗	Q-tips	78914727
Nosecone	Patterson Scientific	NC9854822
Y-splitter, diverter valves, tubing	Patterson Scientific	78914723
Paper tape∗∗	Genesse	88-311A
Latex surgical gloves∗∗	Ansell ENCORE	5711103PF
Towel drape∗∗	Kent Scientific	SURGI50233
O.R. towels∗	Medline	MDT2168284
4-0 vicryl suture∗∗	Med Vet International	J304H
#11 blade∗	Surgical Design	BLADE#11
#15 blade∗	Surgical Design	BLADE#15
Scalple handle∗	Fisher Scientific	12-000-163
Adson tissue forcep∗	Fisher Scientific	13-820-072
Curved pick-ups∗	Excelta	7S
Castroviejo needle holder∗	Fine Science Tools	1256514
Scissors∗	Integra™ Miltex™	V95304
Hemostat∗	Fisher Scientific	12-000-171
Timer∗∗	Fisher Scientific	14-649-17
Empty mouse cage	Vivarium	N/A

∗ indicates critical reagents.

∗∗ indicates common agents that can be obtained from other vendors, or substituted as appropriate.

## Step-by-step method details

### Day before surgery

**Timing: Carprofen cup: 5 min, measurement and group assignment: 30**–**60 min**

1. Ensure surgical area and wide surrounding margin are clear of hair the day prior to surgery. Use clippers to remove additional hair if needed.

***Note:*** Take care not to irritate or break skin barrier with clippers as this may impact experimental outcomes.

2. Place 1 mg carprofen analgesia prepared in Medigel cup in each mouse cage within 24 h of surgery.
3. Measure tumor sizes with caliper and assign groups such that each experimental cohort has an equal average starting tumor volume (troubleshooting 1).

### Setup: Skin preparation area

**Timing: 5 min**

4. To set up the skin preparation area as shown in Figure 1:
  a. Begin by laying down absorbent under pad over prep area.
  b. Place heating pad underneath absorbent pad.
  c. Turn on heating pad and confirm by touch that the surgical field is at an appropriate temperature.
  d. Place 10% betadine solution and 70% ethanol at edge of area with 2×2″ gauze.
  e. Open eye lubrication and Q-tips.

**Figure 1:**
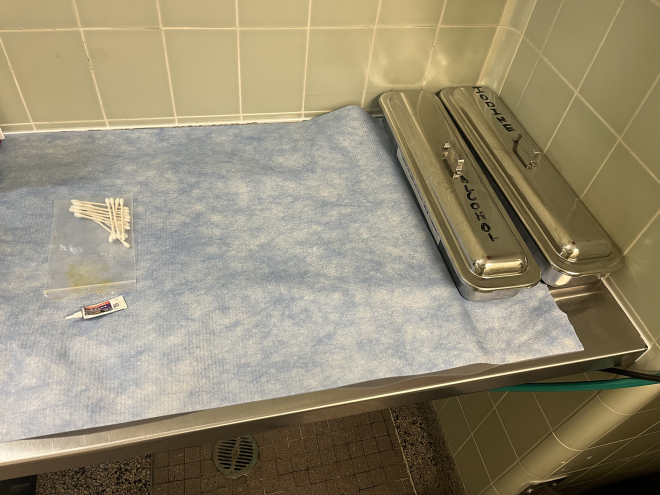
Prep area set up Betadine- and alcohol-soaked gauze (depicted here in silver trays) available for sterilization of mouse flank. Eye lubrication and Q-tips to protect mouse eyes. Nosecone to be placed on absorbent pad for continued mouse anesthesia during prep (not depicted here).
5. Connect tubing from oxygen tank to isoflurane vaporizer, induction chamber, preparation area nosecone, and charcoal canister.
  a. From isoflurane vaporizer, connect a Y-splitter followed by diverter valves such that isoflurane can be directed to induction chamber and/or nosecone.
  b. Ensure one tube from splitter is connected to induction chamber and the other to nosecone.
  c. Connect charcoal canister to induction chamber to scavenge isoflurane.

### Setup: Surgical field

**Timing: 10 min**

6. To set up the sterile surgical field as shown in Figure 2:
  a. Lay down absorbent under pad over prep area and use paper tape to secure the pad in place.
  b. Place heating pad on absorbent under pad and ensure it is positioned such that nosecone (or anticipated mouse location) and surgical working area will be centered on top of the heating pad.
  c. Turn on pad and confirm by touch that the surgical field is at an appropriate temperature.
  d. Turn on overhead surgical light (if available).
  e. Don sterile gloves.
    ***Note:*** At this point the surgeon should maintain sterility and only touch items to be placed in the sterile field.
    ***Note:*** Maintaining sterility throughout set-up and surgical procedure obviates the need for antibiotic administration to mice. Antibiotic administration impacts host microbiome and subsequently anti-tumor immunity.^14^^,^^15^ Therefore, avoidance of antibiotics permits study of the native immune response without microbiota perturbation.
  f. Sterilely open white towel drape (assistant), then place over heating pad (surgeon).
  g. Sterilely open sterile blue O.R. towels (assistant), then grasp towels without touching outside (non-sterile) portion of packaging (surgeon).
  h. Lay down blue O.R. towels to cover entire white drape and working area. Do this by overlapping towels by about 5 to 6 cm.
    ***Note:*** O.R. towel packaging (blue paper) can be used as an additional sterile towel in creation of the sterile field.
    ***Note:*** It is critically important that only one side of the towels contact the table and/or heating pad underneath. In this way the upper side, which contacts the mouse, remains sterile.
  i. Connect tubing to the isoflurane vaporizer but ensure tubing does not contact the sterile field at this point.
  j. Rub 10% betadine soaked 2×2 gauze across entire surface of nosecone, then at least 20 cm of tubing (assistant).
  k. Lay betadine prepped nosecone and tubing on the sterile blue towels (surgeon).
  l. Ensure nosecone is overlying heating pad and in ergonomically appropriate position to perform operation(s).

**Figure 2:**
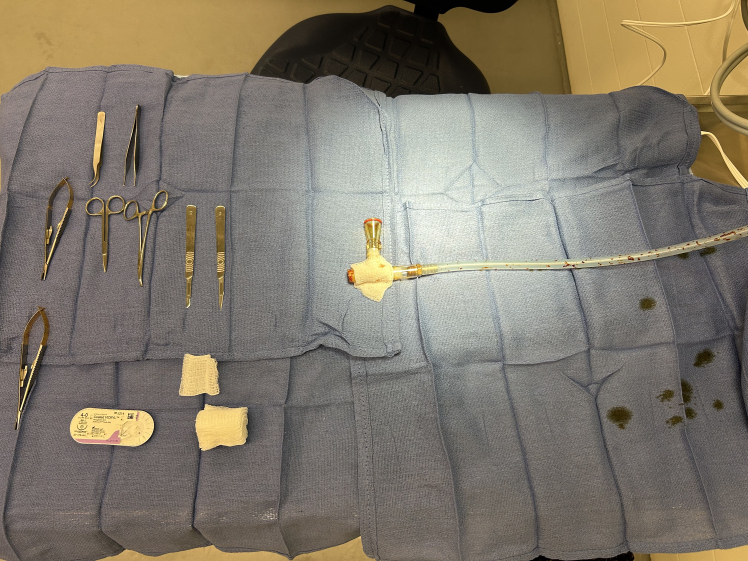
Surgical field set up Sterile blue O.R. towels cover bench to preserve sterile field. Nosecone attached to isoflurane vaporizer, coated with betadine prior to placing in sterile field.
7. Place items in sterile field.
  a. For steps 7b–e, open all item packages in sterile fashion and sterilely drop onto field.
    ***Note:*** Surgeon can break sterility and open items onto the sterile field then don new sterile gloves prior to performing operations, or assistant can sterilely open items and drop them individually onto sterile the field.
  b. Open 4-0 vicryl sutures in sterile fashion and drop onto field, estimating one suture per mouse (additional as needed).
  c. Open one #11 and one #15 blade.
  d. Open two scalpel handles, Adson tissue forceps, curved pick-ups, Castroviejo needle holder, hemostat, scissors, and 2×2 gauze.
  e. Use hemostat to slide blades onto scalpel handles.
    ***Note:*** To avoid injury, pull lower edge (away from cutting edge) over handle. Do not use pushing motion from upper edge or cutting edge.
8. Place timer adjacent to, but not on, sterile field.
  a. Record operating times (assistant).

### Setup: Recovery area

**Timing: <5 min**

9. To set up the recovery area as shown in Figure 3:
  a. Begin by laying down absorbent under pad over prep area.

**Figure 3:**
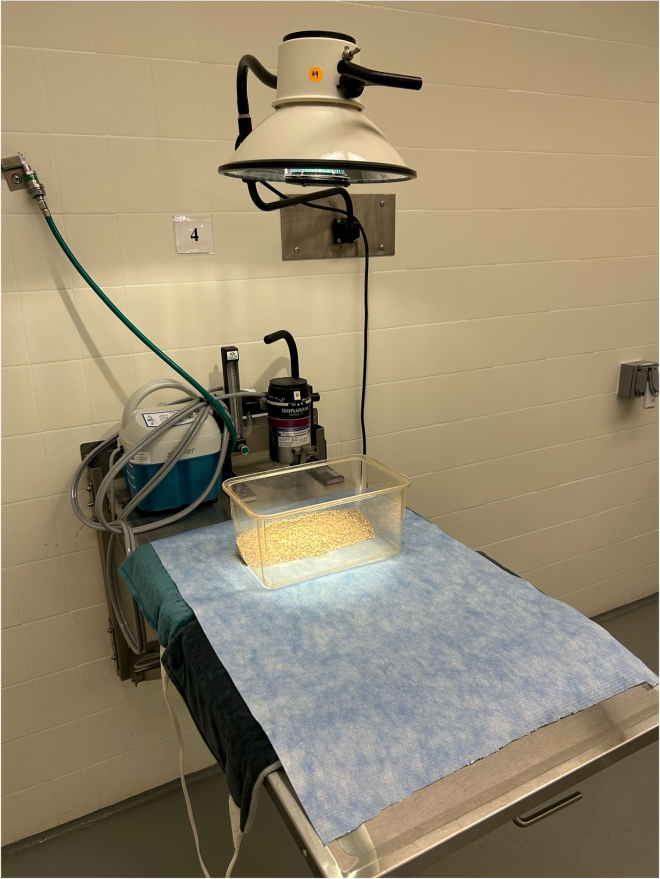
Recovery area set up Mouse cage emptied of all contents (except bedding) placed on heating pads with direct lighting for additional warmth (if available).
10. Place heating pad underneath absorbent pad and ensure recovery cage overlies heating pad.
11. Place new mouse cage, emptied of all contents except bedding, over heating pad.

### Mouse preparation

**Timing: 7**–**10 min per mouse**

12. Steps 13–18 are to be performed by the assistant.
13. Place mouse in induction chamber and begin flow of isoflurane to the chamber.
14. Once mouse is unresponsive, place on prep area absorbent pad with face in the nosecone.
15. Apply liberal eye lubrication to each eye using Q-tip.
16. Prepare skin at the operative site.
  a. Use betadine soaked 2×2 gauze to coat operative site and mouse flank.
  b. Start over operative site and proceed in outward circular fashion.
    i. Repeat steps 16a and 16b for a total of 3 times, using new gauze each time.
  c. Use ethanol soaked 2×2 gauze to coat operative site and mouse flank.
  d. Start over operative site and proceed in outward circular fashion.
    i. Repeat steps 16c–d for a total of 3 times, using new gauze each time.

***Note:*** Chlorhexidine-based scrubs can be used in place of betadine and ethanol for operative site preparation. If using these, allow 2 min for prep to dry.

17. Gently pick up mouse by foreleg and tail and transfers to the surgical field (assistant).
  a. Gently place mouse with prepared, operative site facing up and exposed.
  b. Position mouse directly in front of nosecone.

***Note:*** Assistant must take care not to touch sterile field in the process.

***Note:*** Field is not entirely sterile following contact with mice; however, following these steps minimizes bioburden and avoids the need for peri-operative antibiotics.

18. Begin flow of isoflurane anesthesia to nosecone in surgical field.

### Surgical procedure

**Timing: 18**–**20 min per mouse when starting, with experience this is reduced to 10**–**12 min**

19. Remove excess prep fluid on mouse using dry 2×2 gauze (surgeon).

***Note:*** This helps avoids excessive heat loss from vaporization of residual prep. If a chlorhexidine-based prep was used this step is not necessary.

**Figure 4:**
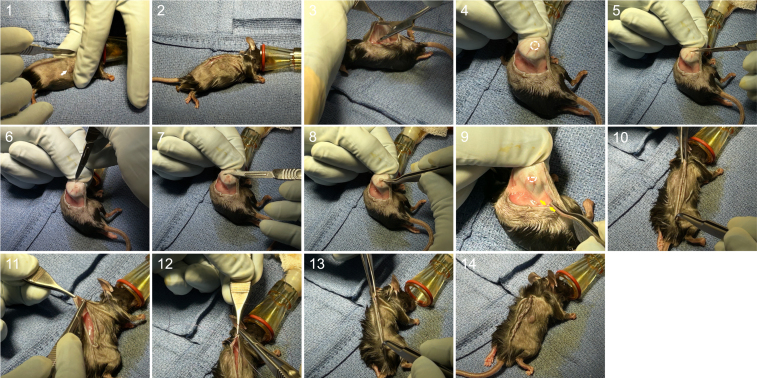
Detailed steps of surgical procedure from left-to-right, top-to-bottom 1. Use index finger and thumb of non-dominant hand to stretch skin (creating tension) perpendicular to planned incision. Tumor indicated by white arrow. 2. Make incision using #15 taking care not to apply too much pressure resulting in cut through underlying tissues. 3. Use hemostat to develop subcutaneous plane. 4. Grasp very edge of skin using index finger and thumb of non-dominant hand. Position index finger under external face of tumor. Lightly supinate hand such that tumor is exposed in the surgical field (tumor outlined in white dashed circle). 5. Bisect tumor using #11 blade with incision oriented perpendicular to an imaginary line from tumor to draining lymph node. 6. Use #11 blade to delicately dissect thin connective tissue and vasculature from around half of tumor to be removed. 7. Continue dissection in plane between tumor and dermis to completely undermine distal half of tumor and free from skin. 8. Once adequately dissected, use curved pickups to remove distal tumor half. 9. Pick-ups pointing to inguinal node within fat pad (and bracketed by yellow arrows). Remaining tumor half outlined in white dashes. 10. Lightly pull edges of incision to approximate skin edges. 11. Begin closure by taking bite of either skin edge. Pull suture through until tail is a comfortable length for tying. 12. Reload needle and take bite of opposite skin edge equidistant from incision apex. Bites on both sides of skin should also be equidistant from cut skin edge. 13. Tie suture down with 3 and 4 throws for vicryl or silk sutures. Cut suture, leaving only 1 to 2 mm tails. In one smooth, gentle motion pull edges of incision such that knot “pops” under the skin to bury it. 14. Complete closure by repeating this process placing sutures every 4 to 5 mm.
22. Use Adson forceps in non-dominant hand to pick up skin at the incision edge and on the side harboring the tumor.
23. While holding skin up for visibility, slide hemostat (closed, dominant hand) forward under the skin and perpendicular to underlying tissues (troubleshooting 3).
  a. Spread hemostat slowly such that the skin pulls away from the underlying tissue to develop the subcutaneous plane.
    ***Note:*** Spread such that tines of hemostat are 1–1.5 cm apart.
  b. Withdraw the hemostat in open position.
  c. Repeat this several times or until the entire tumor and surrounding area are adequately exposed.
    ***Note:*** Thin, avascular adhesive bands may develop in the process. These can be cut sparingly to improve tumor exposure.
24. Using non-dominant hand, delicately grab cut skin edge on side harboring tumor with index finger and thumb and evert such that the tumor is now directly overlying the pad of the index finger and is easily palpable.

***Note:*** Take care not to apply undue upward pressure with index finger, as this may make dissection difficult and/or rupture the tumor capsule.

20. Perform toe-pinch to confirm adequate depth of anesthesia prior to making incision.
  ***Note:*** If anesthesia is adequate there will be absence of withdrawal following pinch (ensure foot has been prepped for this purpose or have assistant perform toe-pinch in preparation area).
  a. Periodically monitor for maintenance of appropriate depth of analgesia via assessment of breathing rate, which will be fewer, larger breaths (as opposed to many shallow breaths).
21. Use a #15 blade to make a 2 cm incision over mouse flank. See Figure 4 (troubleshooting 2).
  a. Plan the incision 5–10 mm dorsal to the flank tumor to ensure ease of tissue closure.
  b. Use index finger and thumb of non-dominant hand to gently press and spread mouse skin of upper abdomen/flank such that it is taught.
    ***Note:*** Do not press directly on tumor.
  c. Make planned 2 cm incision with #15 blade.
    ***Note:*** Incision length can vary depending on experimental objectives. However, the length of incisions should be kept constant for each series of experiments.
  d. Deepen incision as needed such that it is full thickness through the mouse skin and pink/red muscle is visible beneath.
    ***Note:*** If portions of the incision are not full thickness, scissors can be used to cut through the path of the incision.

25. Use #11 blade to transect tumor, leaving 50% in place, or however much residual tumor is desired for experimentation (troubleshooting 5).
  a. First, carefully cut tumor surface, such that 50% of tumor will be resected.
    ***Note:*** Percent of tumor resection is estimated as best possible by eye. If more precise measurement is desired, a sterile ruler can be used to better identify where tumor should be incised to remove desired amount.
  b. Using the body of the blade, deepen cut through entire tumor, taking care to reach but not violate the dermis.
    ***Note:*** Orient this cut such that half of tumor distal to the draining lymph node (i.e. inguinal node) is removed. In this fashion, lymphatic drainage to the residual tumor is preserved.
    ***Note:*** Bisecting the tumor is done first because as surrounding connective tissue is released the tumor tissue recoils and it becomes difficult to visualize where to cut for desired amount of tumor removal.
  c. Inspect the tumor for large feeding blood vessels and tie off large vessels prior to further dissection to prevent bleeding.
    i. Use a Castroviejo needle holder to pass 4-0 vicryl suture under vessel (but not entering the dermis), then tie off tightly and cut suture tails.
    ii. Transect the vessel on the tumor-side (“go-side”) of the tie.
      ***Note:*** This approach provides excellent hemostasis but is typically optional, as bleeding can be controlled with pressure.
  d. Use tip of the #11 blade to delicately and circumferentially release connective tissue around portion of tumor to be removed, starting 1 to 2 mm away from tumor.
  e. Use blade body oriented parallel to the dermis, starting 1 to 2 mm away where connective tissue was released, to circumferentially develop a plane between clear connective tissue and dermis around the half of tumor to be removed.
  f. Develop this plane under the tumor until the initial bisecting cut is reached.
    ***Note:*** Take care not to enter dermis during this step. Entering the dermis can be seen as a thin layer of opaque white tissue starting to be pulled up with the tumor. If partial thickness dermis is removed with the tumor, skin vascularity may become compromised, causing skin necrosis.
  g. Once tumor is freed in this fashion, remove tumor and any remaining attachments using curved forceps.
  h. Inspect the site of tumor removal to confirm that all tumor has been dissected off dermis and that a thin layer of tumor is not remaining attached to the dermis.
  i. If tumor is remaining, carefully repeat steps 25e–g.
    i. Inspect surgical field for bleeding. Control any encountered bleeding with light pressure using 2×2 gauze.
26. Inguinal lymph node removal (optional).
  a. Further develop subcutaneous plane toward the hind leg.
    i. Hold skin edge with Adson forceps in non-dominant hand and use hemostat in dominant hand to further develop subcutaneous plane toward the hind leg (as in steps 22 and 23).
      ***Note:*** The inguinal node resides in the subcutaneous adipose tissue adjacent to the hind leg.
  b. Once adipose tissue is sufficiently exposed, use curved forceps in right hand to mobilize and inspect adipose tissue for inguinal lymph node, which is embedded in the fat pad.
    ***Note:*** Gently stretching the most cephalad edge of the adipose tissue can help expose the node. Using a curved forceps in both hands may aid exposure and dissection.
  c. Once node is exposed, use pointed tips of curved forceps to dissect fatty tissue immediately adjacent to the node such that it can be removed with gentle traction.
    ***Note:*** Avoid causing extensive tissue trauma using small, intentional movements to release the lymph node from surrounding tissue.
27. Axillary lymph node removal (optional).
  a. Further develop subcutaneous plane toward foreleg.
    i. Hold skin edge with Adson forceps in non-dominant hand and use hemostat in dominant hand to further develop subcutaneous plane toward the front leg (as in steps 22 and 23). The axillary node resides in the subcutaneous adipose tissue adjacent to the front leg.
      ***Note:*** When approaching the axillary node from a flank incision, as described in this protocol, the adipose tissue containing the axillary node is encountered just prior to reaching the muscular tissue of the front leg.
  b. Once adipose tissue is sufficiently exposed, use curved forceps to mobilize fatty tissue and inspect for the axillary node, which is embedded within this adipose pad.
  c. Once the node is exposed, use curved forceps to dissect adipose tissue peripherally such that node can be removed with gentle traction.
    ***Note:*** Avoid causing extensive tissue trauma using small, intentional movements to release the lymph node from surrounding tissue.
28. Sham surgery (optional).
  a. Make incision, as described in step 21, on contralateral mouse flank (opposite to tumor).
    ***Note:*** Sham incisions should be of equal length to those in the incomplete resection group.
  b. We similarly develop the subcutaneous plane, as described in steps 22 and 23.
  c. Hold mice in anesthesia for similar length of time as incomplete resection group.
29. Close the surgical incision (troubleshooting 4).
  a. Load 4-0 vicryl on a tapered needle to Castroviejo needle holder.
  b. Deep dermal or simple interrupted technique can be used. We will describe buried, “deep dermal” sutures.
  c. Pick up skin edge with Adson pick-up in non-dominant hand.
  d. Pass suture needle superficially through small amount of dermis tissue with dominant hand.
    ***Note:*** Take care not to pass needle through entire thickness of dermis.
    ***Note:*** Orient this throw such that dermis is entered further from the incision and exited closer to the incision (“deep-to-superficial”).
  e. Reload the needle driver and use Adson in non-dominant hand to grasp opposite skin edge.
  f. Directly across from the first throw, pass suture needle superficially through small amount of dermis.
    ***Note:*** Take care not to pass needle through entire thickness of dermis.
    ***Note:*** Orient this throw such that dermis is entered closer to incision and exited further from incision (“superficial-to-deep”). The distance between dermis entry and exit as well as that of the suture to the skin edge should be approximately equal on both sides.
  g. Pull suture through to desired length and tie.
  h. After each suture is tied down, gently but swiftly pull incision at each apex (such that tension is parallel to the incision) such that knot “pops” under the incision and is “buried”.
    ***Note:*** In this way, sutures are not accessible to mice and cannot be chewed and/or removed.
  i. Place successive sutures approximately 4 and 5 mm apart for length of the incision.
  j. Inspect the incision to ensure skin is well-approximated over the length of the incision.
30. Figure 4 provides corresponding step-by-step images of the surgical procedure as detailed in steps 21–29.
31. Pick up mouse directly over incision (surgeon) and place in hand of assistant for transport to recovery.
  a. Use index finger and thumb to grasp skin on either side of incision such that there is no tension on the closed incision.

***Note:*** Do not grasp directly on tumor.

***Note:*** Surgeon must take care that hand does not contact any non-sterile surfaces. If this happens, remove gloves and don new sterile gloves.

32. Proceed to next mouse surgery (surgeon).

***Note:*** Following the procedure as described, surgeon’s hands and tools remain sterile, as they only come into contact with prepped areas of the mouse. Therefore, sterile gloves do not need to be changed between individual mouse surgeries unless there is a breach of sterile technique.

***Note:*** If tools come into contact with non-sterile surfaces or tissue, then use a new tool, or re-sterilize tool using a glass bead sterilizer.

33. Ensure all experimental cohorts—no surgery, sham, and incomplete resection—receive identical anesthesia and analgesia exposures.
  a. Place mice in the no surgery cohort in the isoflurane induction chamber for a period equivalent to the average time per mouse surgery.

### Mouse recovery

**Timing: 15**–**20 min**

34. Place post-surgical mouse in recovery cage (assistant).
  a. Place mouse with chest facing down such that incision is not in direct contact with cage or bedding.
35. Ensure heating pad and lamp (or overhead surgical light) are on and that cage is at an appropriately warm temperature.
36. Monitor mice for return of normal respirations and spontaneous movement or ambulation.

***Note:*** Time needed will depend on depth of anesthesia used for surgery.

### Tissue harvest

37. A predetermined time after surgery, sacrifice mice and harvest tissues.
38. Harvest residual tumor and draining lymph node to assess for local effects, as previously described.^16^
39. If desired, harvest the spleen to assess for systemic effects, as previously described.^17^
40. If desired, inspect the lungs and liver to assess for metastatic disease.
41. Tissue processing and cell preparation for flow cytometric analysis has been described in detail elsewhere^18^ and is beyond the scope of this protocol.

## Expected outcomes

Using this protocol, mice bearing tumors in the subcutaneous flank can be reproducibly subjected to surgical trauma with partial tumor resection. Based on our experience, all mice are expected to survive surgery without local or systemic evidence of infection.

**Figure 5:**
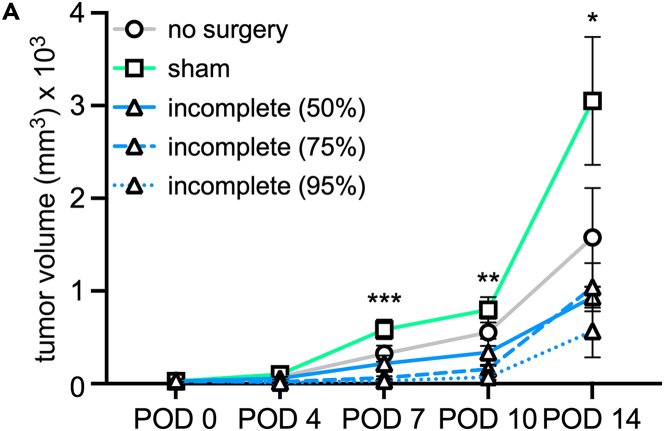
Incomplete oncologic surgery accelerates subsequent regrowth of residual tumors (A) Male C57BL/6J mice bearing TAO1 (murine sarcoma) tumors were subjected to no surgery, sham surgery, or incomplete resection where an estimated 50%, 75%, or 95% of the tumor was removed. Residual tumor growth was then measured by caliper. Sham tumors, exposed to systemic surgical trauma, experienced accelerated growth. Incompletely resected tumors regrew at an accelerated rate. *n* = 3–4 mice per group. Significance determined by one-way ANOVA, ∗*p* < 0.05, ∗∗*p* < 0.005, ∗∗∗*p* < 0.0005.

Illustrating expected outcomes, wild-type B6 mice bearing the syngeneic murine sarcoma cell line TAO1^13^ were subjected to surgical trauma or not. Tumor size was then successively measured after surgery. When executed as described, tumors in the sham group will grow at an accelerated rate relative to no surgery controls. Incompletely resected tumors will also grow at an accelerated rate. By post-operative day 7 (POD7), there is no significant difference in size of 50% resected tumors and those undergoing no surgery. Similar trends are observed in the 75% and 95% resected cohorts (Figure 5A).

This protocol provides a reproducible approach to study the impact of surgical trauma on a residual primary tumor. Further work using this protocol is anticipated to yield mechanistic insights into changes in immune surveillance and tumor biology incurred by surgical trauma.

## Quantification and statistical analysis

### Tumor measurements

Measure subcutaneous flank tumor growth by caliper. The longest tumor dimension is designated as length (L) and the perpendicular dimension is measured as width (W). Calculate volume using the formula V = 0.4 × L × (W^2^). Perform surgery once tumors reach an average size of 4 to 5 mm diameter (longest dimension). Hold average tumor size at experiment start as consistent as possible across experiments, as variability here can impact experimental outcomes. After survival surgery, measure tumor size by caliper at defined intervals or time points. We recommend waiting 48 h after surgery to begin measurements, as post-operative swelling may obscure tumor size.

### Tissue harvest

Sacrifice mice and harvest mouse tissues—tumor, draining lymph node, spleen, etc.—at a defined experimental time point for further analysis. Protocols for tissue harvest and processing have previously been published (listed above).

### Statistical analysis

The appropriate statistical test will depend on experimental design and pre-determined measured outcomes.

## Limitations

This method can be used for study of metastases development as well; however, regrowth of the residual primary tumor will limit the experiment duration, owing to procedural mouse sacrifice once tumor reaches 15 mm (institution specific). The protocol described herein is specific to subcutaneous flank tumors; however, the techniques involved can be adapted to tumors at other anatomic locations.

Application of this protocol to different murine tumor models will require development of reliable tumor growth curves for each cell line. In this way, approximate durations for tumors to reach a certain size prior to surgery and then regrowth can be used for experimental planning. Consistency of tumors established from different cell lines may impact the technical difficulty of this approach. For example, B16F10 tumors (for which we have similarly implemented this protocol) are highly friable. However, the same protocol can be used, paying attention to: during tumor exposure do not put excessive tension on the tissue as this can disrupt the tumor capsule rendering subsequent tumor bisection less reliable (step 24); the initial cut to bisect the tumor must be done in one smooth motion, carrying the incision down to the dermis, such that if tumor spills out of the incision half of the tumor can still be reliably removed by developing a plane between tumor and dermis and carrying that to the point of bisection (as described in step 25).

## Troubleshooting

### Problem 1

Tumors are not the same size at experiment start (Step 3).

### Potential solution

• Wait until tumors reach a predetermined size prior to start of experimentation, e.g., 4 to 5 mm diameter.
• Assign mice to experimental groups such that each group has the same average tumor size at experiment start.
• Hold average tumor size at experiment start constant across experiments, as different starting sizes can impact outcomes.

### Problem 2

Incisions are not all the same length (Step 21).

### Potential solution

• If multiple individuals will be performing surgeries, ensure consistent technique. For example, different extents of surgical trauma will be expected to differentially impact experiment outcome.^19^
• Hold incision placement constant to assist in standardization.
• Consistent surgical technique is paramount for reproducibility in this model.

### Problem 3

Potential disruption of lymphatic drainage during tumor resection (Step 23).

### Potential solution

• Removal of the tumor draining lymph node (inguinal and/or axillary, pending tumor location on flank) results in fewer lymphocytes trafficking to the tumor.
• Disruption of draining lymphatics may also reduce tumor-specific lymphocytes. Avoid extensive dissection/lysis of clear fibrous tissue while developing subcutaneous plane for tumor exposure. Lyse only adhesive bands necessary for tumor exposure.
• Minimize disruption of peri-tumoral tissue, particularly between tumor and draining lymph node, to preserve lymphatic drainage. Remove only tissue surrounding portion of tumor to be resected unless otherwise desired for experimentation.
• This protocol describes removal of the tumor half distal to the draining inguinal lymph node such that lymphatics draining the residual tumor are preserved.

### Problem 4

Dehiscence of the surgical wound (Step 29).

### Potential solution

• Sutures are buried (as described) such that mice cannot reach them and cause wound dehiscence.
• If mice are fighting, then proceed to single house mice. Fighting mice can open surgical wounds and cause large dehiscence.
• If wound dehisces more than 2 to 3 mm it may be advisable to remove mouse from experiment.
• If frequent wound dehiscence is encountered: (i) distance between successive sutures can be reduced, (ii) ensure knots are tied down tightly and do not come undone (assess a dehisced wound for the presence of undone suture), (iii) ensure knot is not cut when cutting suture tails.
• If an alternative method of wound closure is desired, wound adhesive or wound clips can be employed; however, in our hands, sutures provide the most reliable wound closure.
• Mice should be monitored daily post-operatively for signs of wound dehiscence and appropriate supportive care provided (or mouse removed from experiment, as above).

### Problem 5

Tumor consistency is friable and renders bisection difficult (Step 25).

### Potential solution

• Following the initial tumor bisection, curved forceps can be used to remove the bulk of the tumor.
• Insert one tine in the bisection incision, the other outside the tumor capsule on the “go side”, closing forceps around the half of tumor to be removed, and pulling off friable tumor.
• Proceed to develop a plane between any residual tumor not removed by forceps and dermis.

## Resource availability

### Lead contact

General inquiries can be directed to the lead contact, Joseph G. Crompton (jcrompton@mednet.ucla.edu).

### Technical contact

Questions regarding technical aspects of the described procedure and anticipated outcomes can be directed to the technical contact, Tyler R. McCaw (tmccaw@mednet.ucla.edu).

### Materials availability

No materials that were not previously commercially available were generated during the production of this protocol.

### Data and code availability

No informatics data or unique code was generated during the production of this protocol.

## Acknowledgments

This study was supported by funding from the 10.13039/100000054National Cancer Institute Early-Stage Surgeon Scientist Program supplement 3P30CA016042-49S1 (J.G.C.), the 10.13039/100005595UCLA 10.13039/100008623Broad Stem Cell Research Center Training Program (T.R.M.), the 10.13039/100005595UCLA 10.13039/100007186Jonsson Comprehensive Cancer Center, the 10.13039/100005595UCLA Sarcoma Program, the 10.13039/100005301American College of Surgeons (T.R.M.), the 10.13039/100003341Sarcoma Foundation of America (T.R.M. and J.G.C.), and the 10.13039/100009848Tower Cancer Research Foundation (T.R.M. and J.G.C.). The graphical abstract was created using Biorender.com.

## Author contributions

Conceptualization, methodology, investigation, funding acquisition, and writing – original draft, T.R.M.; methodology and investigation, H.B.D., S.Y.L., S.A.F., and M.S.S.; conceptualization and funding acquisition, F.C.E.; conceptualization, funding acquisition, and writing – review and editing, J.G.C.

## Declaration of interests

The authors declare no competing interests.
